# Supplementary material for: Regulatory Roles of Long Non-Coding RNAs Relevant to Antioxidant Enzymes and Immune Responses of Apis cerana Larvae Following Ascosphaera apis Invasion
Source: Int J Mol Sci. 2023 Sep 16;24(18):14175. doi: 10.3390/ijms241814175 (PMC10532054; doi:10.3390/ijms241814175)
Supplement: Supplementary file 1 [file ijms-24-14175-s001.zip › Figure S1.pdf]

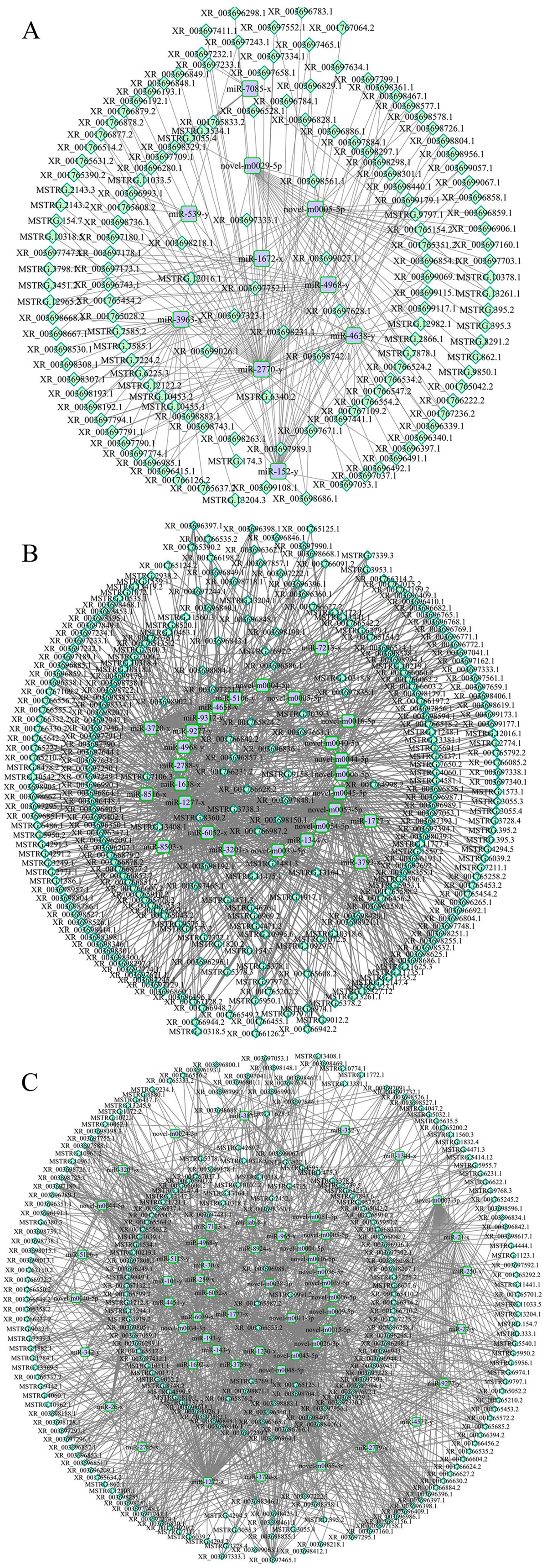

Figure S1. DElncRNA-DEmiRNA regulatory networks in the 4- (A), 5- (B), and 6-day-old (C) comparison groups. The diamonds represent DElncRNAs and the triangles represent DEmiRNAs.
