# Supplementary material for: Regulatory Roles of Long Non-Coding RNAs Relevant to Antioxidant Enzymes and Immune Responses of Apis cerana Larvae Following Ascosphaera apis Invasion
Source: Int J Mol Sci. 2023 Sep 16;24(18):14175. doi: 10.3390/ijms241814175 (PMC10532054; doi:10.3390/ijms241814175)
Supplement: Supplementary file 1 [file ijms-24-14175-s001.zip › Figure S2.pdf]

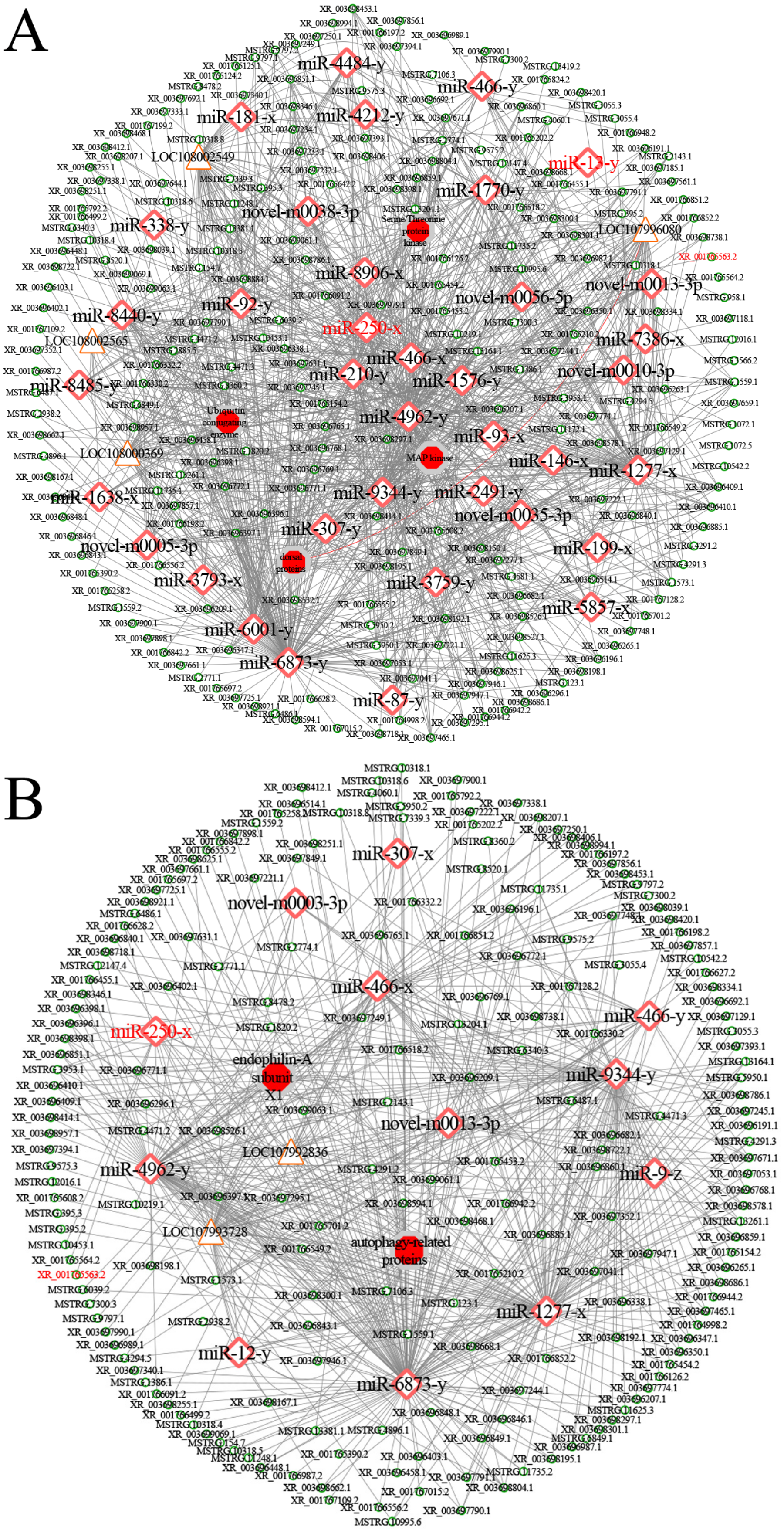
A
miR-4484-y
miR-466-y
miR-181-x
miR-4212-y
miR-13-y
LOC108002549
miR-1770-y
novel-m0038-3p
Serine/Threonine protein kinase
miR-338-y
LOC107996080
miR-8906-x
novel-m0056-5p
novel-m0013-3p
miR-92-y
miR-8440-y
miR-250-x
miR-7386-x
LOC108002565
miR-466-x
novel-m0010-3p
miR-210-y
miR-1576-y
miR-8485-y
Ubiquitin conjugating enzyme
miR-4962-y
miR-93-x
LOC108000369
MAP kinase
miR-146-x
miR-1277-x
miR-1638-x
miR-9344-y
miR-2491-y
novel-m0035-3p
miR-307-y
novel-m0005-3p
dorsal proteins
miR-199-x
miR-3793-x
miR-3759-y
miR-5857-x
miR-6001-y
miR-6873-y
miR-87-y
B
miR-307-x
novel-m0003-3p
miR-466-x
miR-466-y
miR-250-x
endophilin-A subunit X1
miR-9344-y
novel-m0013-3p
LOC107992836
miR-4962-y
miR-9-z
LOC107993728
autophagy-related proteins
miR-1277-x
miR-12-y
miR-6873-y

Figure S2 DElncRNA-involved ceRNA regulatory network relevant to cellular and humoral immune in the *A. c. cerana* 6-day-old larval gut. Diamonds represent miRNAs, circles represent lncRNAs, triangles represent mRNAs, and hexagons represent proteins.
