# Supplementary material for: Regulatory Roles of Long Non-Coding RNAs Relevant to Antioxidant Enzymes and Immune Responses of Apis cerana Larvae Following Ascosphaera apis Invasion
Source: Int J Mol Sci. 2023 Sep 16;24(18):14175. doi: 10.3390/ijms241814175 (PMC10532054; doi:10.3390/ijms241814175)
Supplement: Supplementary file 1 [file ijms-24-14175-s001.zip › Figure S3.pdf]

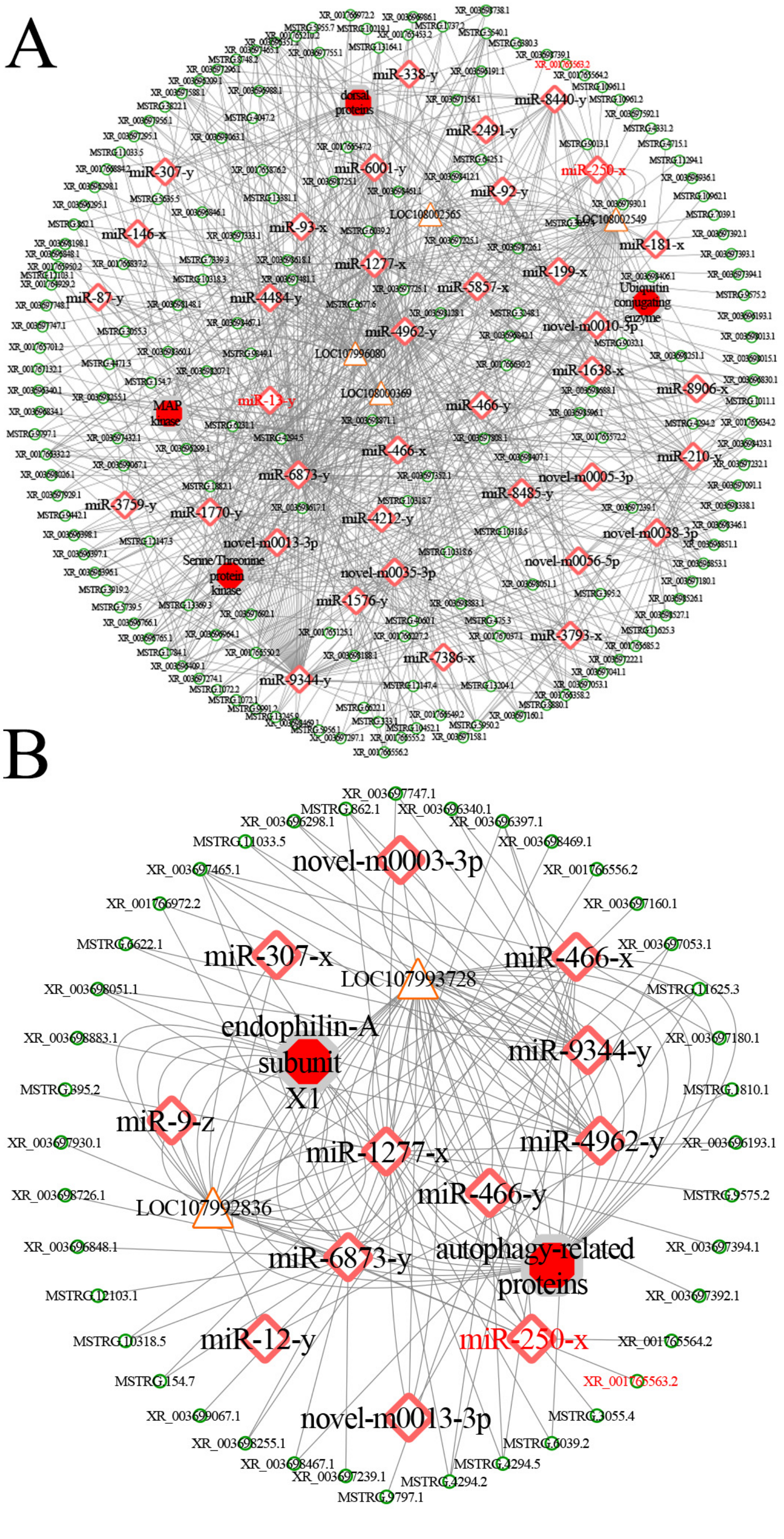

Figure S3. 10 DElncRNA-involved ceRNA regulatory network relevant to cellular and humoral immune in the *A. c. cerana* 6-day-old larval gut. Diamonds represent miRNAs, circles represent lncRNAs, triangles represent mRNAs, and hexagons represent proteins.
