# Supplementary material for: Regulatory Roles of Long Non-Coding RNAs Relevant to Antioxidant Enzymes and Immune Responses of Apis cerana Larvae Following Ascosphaera apis Invasion
Source: Int J Mol Sci. 2023 Sep 16;24(18):14175. doi: 10.3390/ijms241814175 (PMC10532054; doi:10.3390/ijms241814175)
Supplement: Supplementary file 1 [file ijms-24-14175-s001.zip › Table S1 .pdf]

Table S1 Primers used in this chapter

Name	Sequence
XR_003697465.1-F	AAGAATCACGGCACATACA
XR_003697465.1-R	GGATAGAATTGGAAGCATTGG
MSTRG.1072.5-F	CACTTGTTACGACCTGTTCT
MSTRG.1072.5-R	CGACCGTCCATTCTCTGA
XR_003697582.1-F	CGTTGTTGGATGGTGGAA
XR_003697582.1-R	CGATGGTGAGCGTAGAAG
XR_001765834.2-F	AGAAACGGGAGGACGAAT
XR_001765834.2-R	CATCACACGACCAACCAT
MSTRG.9797.1-F	GAGGAGAGGTAGCGAGAA
MSTRG.9797.1-R	CGAAGCAAGGACAGGTAG
XR_001765563.2-F	CGGCATTGGAATCATTAGG
XR_001765563.2-R	TCATCTTAGTTGCTCGTGTA
XR_001766844.2-F	GGAGACCAGGATAATTGACT
XR_001766844.2-R	GACACGACTACACGAAGG
XR_003696857.1-F	CAACTCTTCGCTTATCTCTAC
XR_003696857.1-R	TTCTTCTTCGCTTGGACTT
XR_001765043.2-F	AATACAGCGGTGAAGGAAT
XR_001765043.2-R	TTGGCAGGTTGATGAGATT
XR_003696191.1-F	TTATCCTCTTCCTCTCATTGC
XR_003696191.1-R	ACCGCTTATTATCTCTGTCTC
XR_003697158.1-F	GGTCGTTAGGTCGTTCTG
XR_003697158.1-R	GCGTGATAATGCGTGTTC
XR_003697274.1-F	GCGTCTACAAGCGGAAT
XR_003697274.1-R	CTCACTGGTGGCGTTAG
MSTRG.13245.9-F	CTGATTGGCGAATGATAGC
MSTRG.13245.9-R	GTCCACCTACAACAAGAGT
XR_003696193.1-F	TACACTTGATACGACGCATA
XR_003696193.1-R	GTTCACGCTCTGTCTCTT
actin-F	TTATATGCCAACACTGTCCTTT
actin-R	AGAATTGATCCACCAATCCA
